# Association of neurocognitive disorders with morbidity and mortality in older adults undergoing major surgery in the USA: a retrospective, population-based, cohort study

**DOI:** 10.1016/S2666-7568(23)00194-0

**Published:** 2023-11

**Authors:** Alexander T Abess, Stacie G Deiner, Alexandra Briggs, Elizabeth L Whitlock, Kristin E Charette, Vinca W Chow, Shahzad Shaefi, Pablo Martinez-Camblor, Alistair James O’Malley, Myles Dustin Boone

**Affiliations:** **Department of Anesthesiology and Perioperative Medicine** (A T Abess MD, Prof S G Deiner MD, K E Charette MA, V W Chow MD, P Martinez-Camblor PhD, M D Boone MD), **Department of Surgery** (A Briggs MD), **and Department of Neurology** (M D Boone), **Dartmouth Hitchcock Medical Center, Geisel School of Medicine at Dartmouth, Lebanon, NH, USA; Department of Anesthesia and Perioperative Care, University of California, San Francisco School of Medicine, San Francisco, CA, USA** (E L Whitlock MD); **Department of Anesthesia, Critical Care and Pain Medicine, Beth Israel Deaconess Medical Center, Boston, MA, USA** (S Shaefi MD); **Department of Biomedical Data Science, The Dartmouth Institute for Health Policy and Clinical Practice, Dartmouth Hitchcock Medical Center, Geisel School of Medicine at Dartmouth, Lebanon, NH, USA** (P Martinez-Camblor, Prof A J O’Malley PhD)

## Abstract

**Background:**

Neurocognitive disorders become increasingly common as patients age, and increasing numbers of surgical interventions are done on older patients. The aim of this study was to understand the clinical characteristics and outcomes of surgical patients with neurocognitive disorders in the USA in order to guide future targeted interventions for better care.

**Methods:**

This retrospective cohort study used claims data for US Medicare beneficiaries aged 65 years and older with a record of inpatient admission for a major diagnostic or therapeutic surgical procedure between Jan 1, 2017, and Dec 31, 2018. Data were retrieved through a data use agreement between Dartmouth Hitchcock Medical Center and US Centers for Medicare and Medicaid Services via the Research Data Assistance Center. The exposure of interest was the presence of a pre-existing neurocognitive disorder as defined by diagnostic code within 3 years of index hospital admission. The primary outcome was mortality at 30 days, 90 days, and 365 days from date of surgery among all patients with available data.

**Findings:**

Among 5 263 264 Medicare patients who underwent a major surgical procedure, 767 830 (14·59%) had a pre-existing neurocognitive disorder and 4 495 434 (85·41%) had no pre-existing neurocognitive disorder. Adjusting for demographic factors and comorbidities, patients with a neurocognitive disorder had higher 30-day (hazard ratio 1·24 [95% CI 1·23–1·25]; p<0·0001), 90-day (1·25 [1·24–1·26]; p<0·0001), and 365-day mortality (1·25 [1·25–1·26]; p<0·0001) compared with patients without a neurocognitive disorder.

**Interpretation:**

Our findings suggest that the presence of a neurocognitive disorder is independently associated with an increased risk of mortality. Identification of a neurocognitive disorder before surgery can help clinicians to better disclose risks and plan for patient care after hospital discharge.

**Funding:**

Department of Anesthesiology and Perioperative Medicine at Dartmouth Hitchcock Medical Center.

## Introduction

Ageing populations are projected to strain health-care systems globally. Approximately one-third of all surgical procedures performed annually in the USA are in patients older than 65 years^1^ and, as the population ages, this proportion is expected to increase. Neurocognitive disorders become increasingly prevalent with age;^2–4^ consequently, a substantial number of surgical patients will present with coexisting neurocognitive disorders.^5^ Neurocognitive disorder is a broad term encompassing various diagnoses that manifest as cognitive function impairment and range from subtle, mild cognitive impairments to severe, disabling dementias.

Pre-existing neurocognitive disorder has been associated with higher postoperative complication rates, increased likelihood of discharge to facilities rather than home, and elevated mortality.^6–8^ However, the generalisability of previous studies is limited because of small study populations, short-term follow-up periods, or focus on specific surgery types or subcategories of neurocognitive disorder; a population-based, comprehensive view of the effect of neurocognitive disorder on a range of surgical outcomes is missing. To address this knowledge gap, we used the US Medicare Database, a federally funded US health insurance programme, to assess mortality and perioperative complications among adults aged 65 years and older undergoing major surgery. In 2021, approximately 60 million US residents (about 18% of the total US population at the time) were enrolled in Medicare. Medicare expenditure in the USA in 2021 exceeded US$900 billion, which is a substantial portion of the total $4 trillion spent on US health care altogether.^9^ In addition to mortality and complications, we aimed to describe the types of surgery and hospital and patient characteristics in this cohort. We hypothesised that the presence of a neurocognitive disorder would be associated with increased mortality and higher incidence of complications in older adults undergoing major surgery. Characterising the surgeries these patients undergo and their postoperative complications might support the design of future quality initiatives and research studies aimed at improving perioperative outcomes.

## Methods

### Study design and participants

This was a retrospective cohort study using claims data for US Medicare beneficiaries aged 65 years and older with an inpatient admission for a major diagnostic or therapeutic surgical procedure between Jan 1, 2017, and Dec 31, 2018. For patients with multiple admissions during this time, the first inpatient event was selected as the index admission. Procedural codes for major diagnostic or therapeutic procedures were determined using Healthcare Cost and Utilization Project methodology.^10^

All analyses adhered to a data use agreement with the Center for Medicare and Medicaid (CMS) through the Research Data Assistance Center at the University of Minnesota and the Committee for the Protection of Human Subjects at Dartmouth Hitchcock Medical Center. The Dartmouth Health Institutional Review Board approved this study, including waiver of participant consent in virtue of the nature of the study (secondary research on data previously collected; institutional review board identification: STUDY 02001078, notification letter dated June 16, 2021). The study was reported following the STROBE reporting guidelines.^11^ We accessed CMS data files dated Jan 1, 2014, to Dec 31, 2019, which included the 100% sample Medicare provider analysis and review file, outpatient, carrier, and master beneficiary summary files. All included patients were required to have available data for 3 years before admission (for assessment of the presence of neurocognitive disorder) and 1 year after admission (for observation of outcomes); for example, a patient with an index surgical hospital admission in 2017 required data from 2014 to 2018.

### Procedures

Neurocognitive disorders are generally subdivided into major and minor classifications on the basis of their relative effect on an individual’s functional status. Major neurocognitive disorder results in impairment of independent living and function, whereas minor neurocognitive disorder does not generally affect independence. Dementia is the term most commonly associated with major neurocognitive disorder, whereas mild cognitive impairment is frequently interchangeable with minor neurocognitive disorder. Specific diagnostic criteria are enumerated elsewhere.^12^ Patients were classified as having a pre-existing neurocognitive disorder according to diagnostic codes including codes by the Diagnostic and Statistical Manual of Mental Illness, 5th edition, and code lists recommended by the Alzheimer’s Association Expert Task Force Consensus Statement and the European Delirium Association and American Delirium Society.^13–16^ These neurocognitive disorders included various dementias, cognitive impairments, delirium, and altered mental status. We chose to include altered mental status because it has been shown to commonly overlap with delirium in the older patient population.^17,18^ On the basis of these diagnoses, we used International Classification of Diseases (ICD) 9th revision and 10th revision codes to identify beneficiaries with a neurocognitive disorder.^19,20^ The transition in usage from ICD-9 to ICD-10 was mandated in the USA on Oct 1, 2015. The complete list of ICD codes used to assemble the neurocognitive disorder cohort is included in the appendix (p 1). To maximise sensitivity, we performed a 3-year look-back from the time of the index admission to identify neurocognitive disorder codes in line with previous studies.^21–23^

The hierarchical condition category, a risk-adjustment model used to estimate future health-care expenditures and provide a numeric summary of a patient’s comorbid conditions, was used to determine baseline health status. Other comorbidities (eg, BMI of 30 kg/m^2^ or above and cancer) and admission source (ie, home, emergency department, skilled nursing facility, or other institution) were also collected. Hospital characteristics (eg, type, size, and region) were recorded. Demographic factors such as age, sex, and race were collected. We chose the Social Deprivation Index (SDI) as a surrogate for socioeconomic status. The SDI is a geographical-based measure that incorporates data from seven categories of socioeconomic factors, such as proportion of people with income below the poverty level, proportion of people with less than 12 years of education, proportion of people living in high-density housing, proportion of people without a vehicle, and proportion of unemployed people. The measure has a direct, linear, positive relationship with social deprivation (in other words, as deprivation increases, so does the SDI score, which ranges from 1 to 100). SDI has been found to correlate with access to various health-care services and readmission rates in cognitive impairment research.^24–27^

Outcomes data were collected from the same CMS files, which include information such as date of death (if applicable), length of hospital stay, and discharge diagnoses. Mortality was obtained from the CMS master beneficiary summary file, length of stay and hospital complications were obtained from the CMS MedPAR file (inpatient data), and pre-existing neurocognitive disorder diagnoses were identified using CMS outpatient and carrier files.

### Outcomes

The primary outcome was overall survival at 30 days, 90 days, and 365 days from index surgery. Secondary outcomes included discharge destination (eg, home, rehabilitation, or skilled nursing facilities), readmission rates, complications (eg, delirium, cardiovascular events [myocardial infarction, pulmonary embolism, stroke, and deep vein thrombosis], infection [pneumonia, urinary tract infection, superficial site infection, wound infection, and sepsis], reoperation, and renal insufficiency. We also described as a prespecified secondary outcome the types of surgery that patients with neurocognitive disorder underwent compared with patients without neurocognitive disorder as well as hospital type and region between the two groups.

### Statistical analysis

Descriptive statistics of the data were collected. The distributions of categorical data were presented as frequencies and proportions. The strength of the association between neurocognitive disorder and the primary and secondary outcomes was measured as hazard ratios (HRs) when the outcome was time-dependent (ie, time-to-death and time-to-readmission), odds ratios (ORs) when the outcome was binary, and as regression coefficients interpreted as differences in expected mean outcomes for continuous variables. The probability of death within the first year was estimated using the Kaplan-Meier estimator. For statistical inferential summaries, we provide the unadjusted and adjusted coefficients with 95% CIs. Different models are provided to represent how the covariates modify the association of neurocognitive disorder with the outcome of interest. We chose three different models to explore the effect of different covariates on the level of association between neurocognitive disorder and mortality: an unadjusted model, model one (adjusting for age, sex, and race), and model two (adjusting for age, sex, race, hierarchical condition category, and SDI). Secondary outcomes were evaluated with a model that was adjusted for demographic variables (ie, age, sex, race, and SDI) and patient variables (ie, BMI of 30 kg/m^2^ or above, history of cancer, hierarchical condition category, admission source from nursing facility, and emergency admission type). Multiple-comparison Bonferroni correction was applied on the CIs for the secondary outcomes. All analyses were performed using R statistical software, version 4.0.1, with two-sided p<0·05 considered statistically significant.

### Role of the funding source

The funder of the study had no role in study design, data collection, data analysis, data interpretation, or writing of the report.

## Results

**Figure 1:**
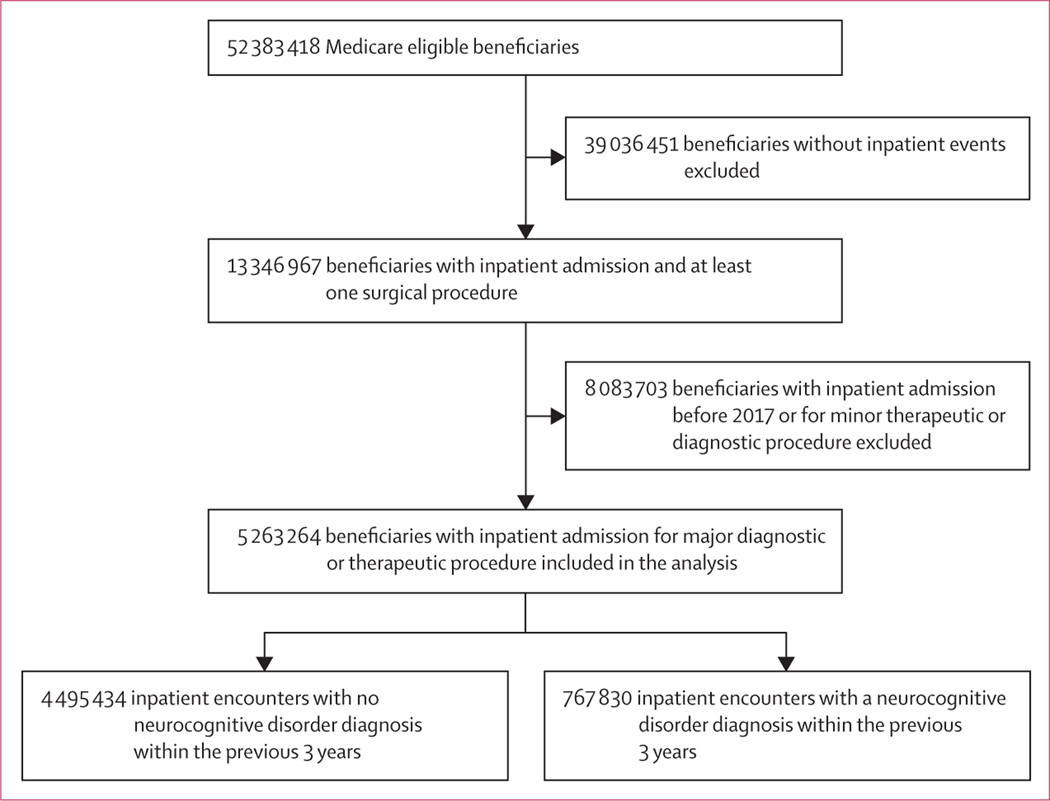
Inclusion flow diagram

Between Jan 1, 2017, and Dec 31, 2018, 5 263 264 eligible patients were admitted to hospital as inpatients for a major diagnostic or therapeutic surgical procedure (figure 1). Of these patients, 767 830 (14·59%) had at least one neurocognitive disorder diagnosis: 413 413 (53·84%) had a single diagnosis, 154 485 (20·12%) had two diagnoses (eg, Alzheimer’s disease with late onset in addition to dementia without behavioural disturbance), 79 365 (10·34%) had three diagnoses, and 120 567 (15·70%) had four or more diagnoses. Thus, the total number of neurocognitive disorder diagnoses exceeded the number of patients with a neurocognitive disorder diagnosis (appendix p 2). Baseline characteristics of the cohort are shown in table 1. Patients with a neurocognitive disorder diagnosis tended to be older and were more likely to be female than those without a neurocognitive disorder. Median hierarchical condition category risk score and SDI were higher in patients with a neurocognitive disorder than in those without a neurocognitive disorder. A greater proportion of patients with neurocognitive disorder were admitted from a skilled nursing facility or through the emergency department, compared with patients without a neurocognitive disorder.

**Figure 2:**
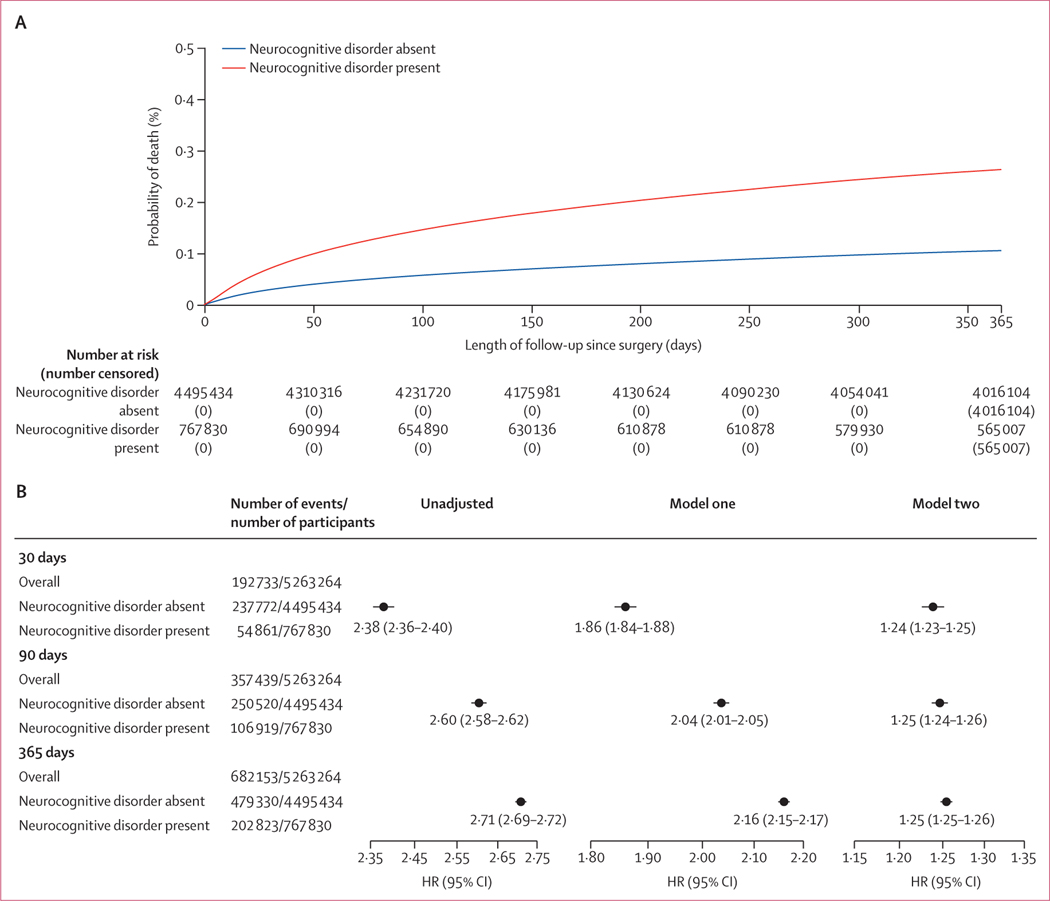
Mortality risk over time and HRs at 30 days, 90 days, and 365 days for unadjusted and adjusted models (A) Kaplan-Meier curve of observed mortality during follow-up in patients with a neurocognitive disorder and patients without a neurocognitive disorder. (B) Forest plots of HRs in the unadjusted and adjusted models for mortality at 30 days, 90 days, and 365 days of follow-up in patients with and without a neurocognitive disorder. Model one is adjusted for age, sex, and race. Model two is adjusted for age, sex, race, hierarchical condition category, and Social Deprivation Index. HR=hazard ratio.

The observed mortality difference between patients with a neurocognitive disorder and patients without a neurocognitive disorder is in figure 2A. Before adjustment, the presence of a neurocognitive disorder compared with no neurocognitive disorder was associated with increased mortality at 30 days (7·16% [95% CI 7·10–7·22] *vs* 3·06% [3·05–3·08], HR 2·38 [95% CI 2·36–2·40]; p<0·0001), 90 days (13·92% [13·85–14·00] *vs* 5·57% [5·55–5·59], 2·60 (2·58–2·62); p<0·0001), and 1 year (26·41% [26·32–26·51] *vs* 10·66% [10·63–10·69], 2·71 [2·70–2·72]; p<0·0001). When adjusting for age, sex, and race (model one), neurocognitive disorder was associated with increased mortality at 30 days (HR 1·86 [95% CI 1·84–1·88]; p<0·0001), 90 days (2·04 [2·01–2·05]; p<0·0001), and 365 days (2·16 [2·15–2·17]; p<0·0001; figure 2B). When adjusting for age, sex, race, hierarchical condition category, and SDI (model two), neurocognitive disorder remained associated with increased mortality at 30 days (HR 1·24 [95% CI 1·23–1·25]; p<0·0001), 90 days (1·25 [1·24–1·26]; p<0·0001), and 1 year (1·25 [1·25–1·26]; p<0·0001; figure 2B).

Adjusted analyses of postoperative complications, readmission rates, and discharge destinations for patients with and without a neurocognitive disorder are shown in table 2. The models were adjusted by age, sex, race, BMI of 30 kg/m^2^ or above, cancer history, hierarchical condition category score, SDI, admission from a nursing facility, and emergency admission. ORs for a diagnosis of delirium, stroke, wound infection, and urinary tract infection during index admission were higher in patients with a neurocognitive disorder than in patients without a neurocognitive disorder (table 2).

**Figure 3:**
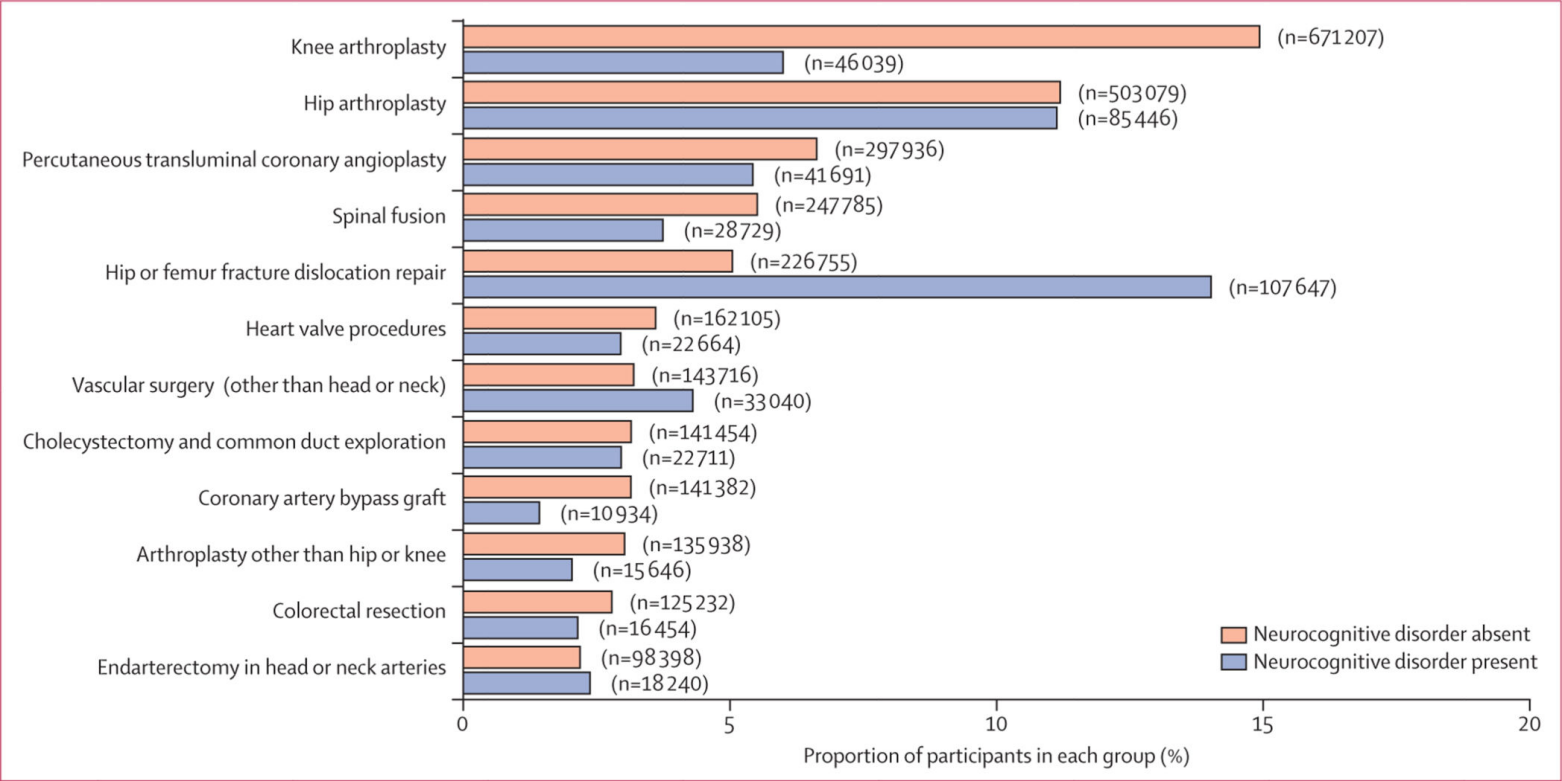
Most frequent surgery types for patients with and without a neurocognitive disorder The figure shows all surgery types that accounted for at least 1% of surgeries. Surgery type is based on the Clinical Classification Software scheme from the Agency for Healthcare Research and Quality.

The five most common surgical procedures for patients with neurocognitive disorder were treatment of a hip or femur fracture (107 647 [14·02%] of 767 830), followed by hip arthroplasty (85 446 [11·13%]), knee arthroplasty (46 039 [6·00%]), percutaneous transluminal coronary angioplasty (41 691 [5·43%]), and vascular surgery (33 040 [4·30%]; figure 3). By comparison, the five most common procedures for patients without neurocognitive disorder were knee arthroplasty (671 207 [14·93%] of 4 495 434), followed by hip arthroplasty (503 079 [11·19%]), percutaneous transluminal coronary angioplasty (297 936 [6·63%]), spinal fusion (247 785 [5·51%]), and treatment of hip or femur fracture (226 755 [5·04%]). Rates for other procedure types were similar across the two groups.

We compared hospital and regional characteristics for patients with and without neurocognitive disorder (appendix p 3). The hospital size and owner type were similar across the two groups. Patients with a neurocognitive disorder were slightly more common than patients without a neurocognitive disorder in rural and southern hospitals. Hospitals outside of the continental USA had fewer patients with a neurocognitive disorder than hospitals in the continental USA (appendix p 3).

## Discussion

In this retrospective cohort study of Medicare beneficiaries aged 65 years or older undergoing major surgical procedures, we observed a significant association between the presence of a neurocognitive disorder and mortality at 30 days, 90 days, and 365 days. These results remained consistent after adjustments for age, comorbidities, and socioeconomic status. Our findings offer population-based estimates of mortality rates in older adults with neurocognitive disorder after undergoing major surgery.

A recent study involving more than 5000 community-living older adults in the USA also showed an increased risk of death after major surgery in patients with dementia and frailty.^28^ Our study substantially extends these findings by showing a similar risk of mortality after major surgery in patients with all types of neurocognitive disorder and across a much larger segment of the US population. In addition, we observed that, compared with patients without a neurocognitive disorder, patients with a neurocognitive disorder had higher rates of perioperative complications such as delirium, stroke, infection, reoperation, and readmission, and were less likely to be discharged to their homes. These different outcomes are likely to be meaningful for care providers, caregivers, and patients, who often prioritise quality of life and functional independence over quantity of life.^29^

An unexpected finding was the lower adjusted risk for some complications (eg, cardiac arrest, myocardial infarction, pulmonary embolism, and superficial site infections) in patients with neurocognitive disorder compared with patients without neurocognitive disorder, which we hypothesise might reflect an epiphenomenon in which symptomatic reporting and clinical detection of these complications in perioperative patients with neurocognitive disorder is hindered. Whether this lower rate of observed complications could potentially be a source of increased mortality because of unrealised treatment for unrecognised conditions is unknown, and further investigation in this topic seems warranted.

In this study, 14·59% of Medicare beneficiaries who had a major surgical procedure had one or more neurocognitive disorder diagnoses. These rates of neurocognitive disorder almost certainly represent an underdiagnosis of these conditions. Only 156 154 (2·97%) of the more than 5·2 million patients included in this study had a diagnosis of cognitive impairment. Previous estimates have indicated that the prevalence of cognitive impairment is several times higher in surgical patients older than 65 years than what we observed in this study.^6,30^ CMS has implemented a recommendation that older patients be assessed for cognitive impairment as part of an annual wellness visit.^31^ Regrettably, this potential benefit remains underused: only 20% of Medicare fee-for-service beneficiaries received a screening examination in 2016, suggesting that older adults who are eligible for cognitive testing via this mechanism continue to be missed.^32^ Beyond issues of accessibility, the effectiveness of the Medicare annual wellness visit in detecting cognitive impairment has been scrutinised.^33^ CMS recommends that providers detect cognitive impairment during the annual wellness visit on the basis of direct observation or any concerns expressed by patients or their family or friends. However, CMS does not endorse the use of a specific screening tool.^31^ Furthermore, in 2020 the US Preventive Services Task Force reiterated its previous finding that the evidence to justify routine cognitive screening in older patients is insufficient.^34^ Their conclusion that there is insufficient evidence to justify routine cognitive screening remains a subject of debate, as the American Academy of Neurology and the Alzheimer’s Association both advocate for the use of a screening tool to assess for cognitive impairment.^35,36^ Uncertainty regarding the relative benefits and harms of cognitive screening arises from the insufficient data describing impact on outcomes that are meaningful to patients, caregivers, and society.^37^

This study suggests the need for preoperative identification of cognitive impairment to inform patient and provider discussions surrounding treatment and recovery. As the global population ages, more adults with neurocognitive disorder will undergo major surgery, highlighting the need to focus efforts on early identification of neurocognitive disorders and perioperative risk mitigation.^38^ The American Society of Anesthesiologists, the European Society of Anaesthesiology, the Royal College of Anaesthetists, the American College of Surgeons, and the American Geriatrics Society all have recommended preoperative cognitive screening for patients older than 65 years before major surgery, but the implementation of these recommendations appears to be limited. This study contributes substantially to the supporting evidence for these recommendations and suggests that collecting intermediate and longer term metrics in patients with neurocognitive impairment will be important to improve care.

Multidisciplinary preoperative programmes aimed at risk stratification of older adults at high risk for perioperative complications have shown success in enhancing postoperative and quality-of-life outcomes.^39,40^ The American College of Surgeons Geriatric Surgery Verification programme, which aims to optimise the surgical care of older adults through implementation of standards spanning both the preoperative and postoperative periods, includes preoperative cognitive assessment as one element of a comprehensive screen for vulnerability.^41^ Implementation of this programme has been shown to reduce the length of inpatient stay.^42^

Delirium prevention strategies have been shown in international settings to effectively reduce the incidence and severity of delirium after surgery.^43^ Our study showed that patients with a neurocognitive disorder had a higher risk of delirium after surgery compared with patients without neurocognitive disorder (OR 1·96 [95% CI 1·91–2·01]), which indicates that this patient population is an important group on which to focus these preventive efforts.

Our study also identified the most common surgical procedures done in older adults in the USA, which might inform targeted subgroup interventions to improve outcomes. For example, we found that fractures are a primary driver of hospitalisations associated with a major procedure in patients with a neurocognitive disorder. Public health prevention programmes designed to reduce fracture risk in older adults might substantially reduce hospitalisation rates.

Our study has several limitations. First, the inherent limitations of administrative claims data must be considered. These data are primarily derived to serve billing and administrative purposes and not clinical research, which might result in inaccuracies or underreporting of specific diagnoses and procedures. Administrative staff examine clinical documentation of procedures and diagnoses performed by clinicians that have varying levels of training (although the billing provider is ultimately responsible for accuracy), and this clinical documentation is then converted to administrative codes. Additionally, the potential exists for a lack of coding granularity, miscoding, or undercoding due to the vast number of codes available, leading to the possibility of misclassification bias or affecting the depth of our analysis. Overarchingly, the retrospective nature of these data with a varied degree of confounding variable data reporting limits the ability to infer causation.

Second, misclassification of the exposure might have occurred. Some patients with a neurocognitive disorder are likely not to have an ICD-coded diagnosis—for example, previous research has suggested that approximately half of all patients with dementia do not have a diagnosis,^44–49^ and using Medicare claims data to identify dementia has been shown to miss about half of clinically diagnosed patients.^22^ As such, our results are likely to be imprecise due to misclassification of patients with a mild neurocognitive disorder in the subgroup of patients deemed not to have a neurocognitive disorder. The degree to which this possible misclassification alters our results is unknown, and potential differences in outcomes might be greater as well as lesser. The presence of falsely classified patients (those with actual disease that evades detection) in the cohort without neurocognitive disorder could cause a relative worsening of outcomes in this cohort (thus minimising the difference between the two groups). The same misclassification could also possibly cause the outcomes to appear worse than reality in the group with a neurocognitive disorder than in the group without a neurocognitive disorder because only the most severe cases of neurocognitive disorder (with potentially the worst outcomes) are currently counted in that group. However, without knowing the actual crossover and effect of the varying degrees of misclassified patients, knowing in which direction or to what degree our results might be skewed is impossible. One of our strategies to increase sensitivity to patients with a presumably undiagnosed mild neurocognitive disorder was to include non-specific diagnoses, such as altered mental status and unspecified signs and symptoms of cognitive function and awareness as neurocognitive disorders. As such, it should be noted that the neurocognitive diagnoses chosen for inclusion are based on ICD codes and are distinct from those described in the perioperative neurocognitive disorder literature.^5^

Third, our ability to examine subtypes of neurocognitive disorder in relation to outcome is limited for several reasons: the transition from ICD 9th revision to ICD 10th revision; the evolving terminology and diagnostic patterns of neurocognitive disorders; and constrained diagnostic nuance, accuracy, and potential duplication from using administrative claims data. For example, we chose not to use the number of neurocognitive disorder diagnoses because the same clinical entity could conceivably be diagnosed with multiple codes (for example, either due to different coding practices or as the disease progresses and changes in presentation). Similarly, we chose not to attempt discrimination between minor and major neurocognitive disorders because the terminology, diagnoses, and coding of neurocognitive disorders has changed substantially over the past 10 years. For these reasons, we deemed it most appropriate to use a dichotomous exposure variable (presence or absence of neurocognitive disorder), which offered a straightforward approach to assess the effect of neurocognitive disorder on our outcomes of interest without the complications that come with grading or stratifying on the basis of the number or severity of diagnoses. This method simplified the interpretation of results, making this interpretation more accessible and providing clear and actionable insights.

Fourth, the composition of our cohort might limit the generalisability of our findings. Medicare claims data are limited to the US population and under-represent patients younger than 65 years, those covered by Medicare Advantage, and those receiving dual Medicare and Medicaid (a state-run insurance plan for younger people, often with low-income, or those with qualifying medical conditions). About 12% of people aged 65 years and older in the USA have Medicare as their sole insurance provider.^9^ Another 40% of US adults older than 65 years also have private insurance, which might be combined with Medicare.^9^ Medicare Advantage programmes are offered by Medicare-approved companies that must follow rules set by Medicare and cover another 40% of US people older than 65 years. However, of note, demographic and socioeconomic characteristics of patients receiving Medicare and Medicare Advantage are similar.^50^

We chose to exclude data from 2020 to 2022 because of disruptions in health-care delivery related to the COVID-19 pandemic. Additionally, we cannot exclude the possibility that unmeasured or incorrectly coded confounding variables affected our modelling. For example, we did not include surgery type as a covariate in our models. Lastly, our 1-year outcome data are limited to mortality.

Future research should be directed towards other patient-centred outcomes such as functional independence and quality of life, which could enhance preoperative decision making. Focused studies for specific surgery types would also be of value, and orthopaedic surgery examination in particular might have a high clinical impact.

Strategies to incorporate neurocognitive disorder screening should be developed so that appropriate risk stratification and supported decision making can occur before surgical interventions. Ideally, neurocognitive disorder screening can occur even in the setting of urgent and emergent procedures to inform preoperative discussions of risk and benefit. Counselling should include the likelihood of skilled nursing admission (at least temporarily) and 1-year mortality, which in many cases providers can tailor to their patient and procedure using tools such as the American College of Surgeons risk calculator.^51^ Identifying the presence of preoperative neurocognitive disorder can inform more accurate supported decision making. Further incorporating the use of patient decision aids has been shown to be associated with several benefits, including more accurate risk perceptions, increased alignment of decisions with patients’ values, reduced internal conflict for patients and families, and decreased pursuit of major elective surgery.^52^

## Supplementary Material

1

## Figures and Tables

**Table 1:** Characteristics of patients with and without a neurocognitive disorder

	Total participants (n=5 263 264)	Neurocognitive disorder absent (n=4 495 434)	Neurocognitive disorder present (n=767 830)
Age, years	74 (69–80)	74 (69–79)	79 (72–86)
Sex			
Female	2 817 877 (53·54%)	2 375 264 (52·83%)	442 613 (57·64%)
Male	2 445 387 (46·46%)	2 120 170 (47·17%)	325 217 (42·36%)
BMI ≥30 kg/m^2^	711 623 (13·52%)	635 205 (14·13%)	76 418 (9·95%)
History of cancer	753 226 (14·31%)	608 374 (13·53%)	144 852 (18·87%)
Hierarchical condition category score	0·97 (0·33–2·26)	0·85 (0·29–1·99)	1·99 (0·97–3·58)
Social Deprivation Index score	40 (19–63)	39 (19–63)	42 (21–66)
Race			
Unknown	79 180 (1·50%)	73 224 (1·63%)	5956 (0·78%)
White	4 520 598 (85·89%)	3 865 690 (85·99%)	654 908 (85·29%)
Black	405 459 (7·70%)	335 815 (7·47%)	69 644 (9·07%)
Other	77 482 (1·47%)	68 380 (1·52%)	9102 (1·19%)
Asian	67 261 (1·28%)	57 138 (1·27%)	10 123 (1·32%)
Hispanic	90 062 (1·71%)	76 102 (1·69%)	13 960 (1·82%)
North American Native	23 222 (0·44%)	19 085 (0·42%)	4137 (0·54%)
Admission from skilled nursing facility	47 268 (0·90%)	18 475 (0·41%)	28 793 (3·75%)
Emergency admission	1705 652 (32·41%)	1 331 196 (29·61%)	374 456 (48·77%)

Data are median (IQR) or n (%).

**Table 2:** Postoperative outcomes in patients with and without a neurocognitive disorder

	Neurocognitive disorder absent (n=4 495 434)	Neurocognitive disorder present (n=767 830)	Adjusted effect size (95% CI)	p value
Post-surgical admission to intensive care unit	1 239 383 (27·57%)	254 281 (33·11%)	0·86 (0·85 to 0·87)	<0·0001
Length of hospital stay, days	3 (2–6)	4 (3–8)	–0·38 (–0·40 to –0·36)	<0·0001
Discharge destination				
Home	2 145 918 (47·72%)	197 293 (25·69%)	0·64 (0·63 to 0·65)	<0·0001
Skilled nursing facility	840 247 (18·69%)	306 379 (39·90%)	1·73 (1·71 to 1·74)	<0·0001
Rehabilitation facility	216 161 (4·81%)	61 551 (8·02%)	1·22 (1·20 to 1·24)	<0·0001
Readmission within 30 days	515 999 (11·48%)	157 998 (20·58%)	1·07 (1·06 to 1·08)	<0·0001
Readmission within 90 days	847 927 (18·86%)	252 956 (32·94%)	1·08 (1·07 to 1·09)	<0·0001
Complications during inpatient stay				
Delirium	41 459 (0·92%)	22 075 (2·87%)	1·96 (1·91 to 2·01)	<0·0001
Pneumonia	110 178 (2·45%)	34 207 (4·46%)	0·93 (0·91 to 0·95)	<0·0001
Pulmonary embolism	23 231 (0·52%)	4445 (0·58%)	0·63 (0·60 to 0·66)	<0·0001
Deep vein thrombosis	53 004 (1·18%)	13 058 (1·70%)	0·79 (0·76 to 0·80)	<0·0001
Stroke	283 789 (6·31%)	80 395 (10·47%)	1·38 (1·36 to 1·40)	<0·0001
Cardiac arrest	26 278 (0·58%)	5993 (0·78%)	0·76 (0·73 to 0·80)	<0·0001
Myocardial infarction	306 990 (6·83%)	44 728 (5·83%)	0·66 (0·64 to 0·66)	<0·0001
Renal insufficiency	1 133 055 (25·20%)	301 110 (39·21%)	1·08 (1·07 to 1·09)	<0·0001
Wound infection	2940 (0·07%)	1190 (0·15%)	1·28 (1·14 to 1·42)	<0·0001
Superficial site infection	21 026 (0·47%)	4255 (0·55%)	0·79 (0·74 to 0·83)	<0·0001
Urinary tract infection	166 145 (3·70%)	69 819 (9·09%)	1·43 (1·40 to 1·45)	<0·0001
Sepsis	118 467 (2·64%)	40 247 (5·24%)	0·93 (0·91 to 1·95)	<0·0001
Need for reoperation	621 (0·01%)	143 (0·02%)	1·19 (0·88 to 1·60)	0·93

Data are n (%) or median (IQR), except where otherwise stated. Effect sizes are adjusted by all covariates reported in table 1. The adjusted effect size for length of stay is reported as the regression coefficient. For readmission rates (within 30 days and 90 days), the effect size is reported as a hazard ratio; all other effect sizes are odds ratios.

## Research in context

### Evidence before this study

Estimates suggest that, by 2030, a sixth of the world’s population will be older than 60 years. One in every three surgeries in the USA is done in patients older than 65 years. Neurocognitive disorders tend to become increasingly common as people age; consequently, increasing numbers of patients requiring surgery are likely to have concurrent neurocognitive conditions. We searched PubMed from Jan 1, 2012 to Dec 31, 2022 for publications in English with the terms “neurocognitive disorder”, “surgery”, and “outcomes” to assess the impact of pre-existing neurocognitive disorders on surgical outcomes. Results were restricted to studies on humans aged 65 years and older, and review articles and meta-analyses were excluded. We found 283 studies, most of which were limited by small numbers of participants and specific surgery types. Surgical patients with a neurocognitive disorder in selected populations have been previously shown to have worse outcomes.

### Added value of this study

This study uses claims data from the US Medicare programme to provide broad, population-based evidence that older patients with pre-existing neurocognitive disorders have a greater risk of mortality and are less likely to be discharged home following major surgery than patients without neurocognitive disorders. This association persists even after adjusting for age, comorbidities, and socioeconomic status. Importantly, this study also provides a description of the common types of major surgery that older patients with neurocognitive disorders undergo, thus highlighting high-yield areas for care improvement efforts.

### Implications of all the available evidence

The presence of a neurocognitive disorder is associated with markedly worse outcomes in older surgical patients. As such, identification of a neurocognitive disorder before surgery is important to allow accurate risk disclosure and care planning at the individual patient level. Ageing populations are projected to strain health-care systems globally. Active screening for neurocognitive disorders might offer an opportunity for health systems to identify particularly vulnerable patients and support efficient deployment of health-care resources.
