# Supplementary material for: Association of neurocognitive disorders with morbidity and mortality in older adults undergoing major surgery in the USA: a retrospective, population-based, cohort study
Source: Lancet Healthy Longev. Author manuscript; Available in PMC 2023 Nov 17. (PMC10654795; doi:10.1016/S2666-7568(23)00194-0)
Supplement: 1 [file NIHMS1942264-supplement-1.pdf]

THE LANCET
Healthy Longevity

Supplementary appendix

This appendix formed part of the original submission and has been peer reviewed. We post it as supplied by the authors.

Supplement to: Abess AT, Deiner SG, Briggs A, et al. Association of neurocognitive disorders with morbidity and mortality in older adults undergoing major surgery in the USA: a retrospective, population-based, cohort study. *Lancet Healthy Longev* 2023; **4:** e608–17.

Appendix Page 1: ICD-10 and ICD-9 codes for Neurocognitive Disorders

NCD Grouping	ICD-10 Code(s)	ICD-9 Code(s)
**Alzheimer's Disease**	G300, G301, G308, G309	3310
**Dementia, unspecified**	F0280, F0281, F0390, F0391	2900-29043 (2900, 29010, 29011, 29012, 29013, 29020, 29021, 2903, 29040, 29041, 29042, 29043, 29410, 29411), 29420, 29421, 2941, 29411, 797
**Vascular Dementia**	F0150, F0151	29040, 29041, 29042, 29043
**Frontotemporal Dementia**	G310, G3109, G3101	33111, 33119
**Lewy Body Dementia**	G3183	33182
**Corticobasal Degeneration**	G3185	3316, 3320
**Other Degenerative and Miscellaneous Dementias**	G311, G319	3312, 3313, 3314, 3315, 3317, 33180, 33181, 33189, 3319, 0461, 0463, 2908, 2909, 3330, 3334
**Cognitive Impairment, unspecified**	R4181, G3184	78093, 33183
**Cognitive Impairment, miscellaneous causes**	I69010-I69019, I69020-I69023, I69028, I69110-I69115, I69118, I69119, I69210-I69215, I69218, I69219, I69310-I69315, I69318, I69319, I69810-I69815, I69818, I69819, I69910-I69915, I69918, I69919,S060, S061, S062, S063, S064, S065, S066, S068, S069, S06.A	4380
**Altered Mental Status**	R4182	78097
**Delirium**	F05, R410	2930, 2931
**Other & Unspecified Signs and Symptoms Involving Cognitive Functions and Awareness**	F068, F688, R419, R414	29389, 3101, 31089, 2948, 2949, 7818, 79951, 79952, 79953, 79954, 79955, 79959, 95901

Appendix Page 2: Types and frequency of Neurocognitive Disorders

NCD Grouping	Number	Percentage of NCD Patients
**Alzheimer's Disease**	123,281	16.1
**Dementia, unspecified**	275,665	35.9
**Vascular Dementia**	54,273	7.07
**Frontotemporal Dementia**	4,698	0.612
**Lewy Body Dementia**	8,539	1.11
**Corticobasal Degeneration**	35,538	4.63
**Other Degenerative and Miscellaneous Dementias**	156,011	20.3
**Cognitive Impairment, unspecified**	156,154	20.3
**Cognitive Impairment, miscellaneous causes**	14,555	1.90
**Altered Mental Status**	379,205	49.4
**Delirium**	54,510	7.10
**Other & Unspecified Symptoms and Signs Involving Cognitive Functions and Awareness**	142,044	18.5
**Total**	1,404,473	

Appendix Page 3: Hospital and regional characteristics of patients with and without NCD

	Total N=5,263,264	NCD Absent N=4,495,434 (85.4%)	NCD Present N= 767,830 (14.6%)
**Academic Medical Center, n (%)**	1,503,169 (28.56 %)	1,293,012 (28.76%)	210,157 (27.37%)
**Beds, mean±sd**	465.6±372.4	466.43±373.97	460.76±363.12
**Owner**			
**Government, n (%)**	620,150 (11.78%)	523,367 (11.64%)	96,783 (12.60%)
**Private, n (%)**	2,758,713 (52.41%)	2,361,191 (52.52%)	397,522 (51.77)
**Religious, n (%)**	552,389 (10.50%)	471,463 (10.49%)	80,926 (10.53%)
**Other, n (%)**	1,332,012 (25.31%)	1,139,413 (25.35%)	192,599 (25.08%)
**Geographic Area, Rural, n (%)**	367,142 (6.98%)	303,614 (6.75%)	63,528 (8.27%)
**Region**			
**Midwest, n (%)**	1,240,518 (23.57%)	1,071,093 (23.82%)	169,425 (22.07%)
**Northeast, n (%)**	940,868 (17.88%)	801,410 (17.83%)	139,458 (18.16%)
**South, n (%)**	2,011,608 (38.22%)	1,682,784 (37.43%)	328,824 (42.83%)
**West, n (%)**	1,020,758 (19.39%)	893,780 (19.88%)	126,978 (16.54%)
**Out of Continental US, n (%)**	49,168 (0.93%)	46,150 (1.03%)	3,018 (0.39%)
**Unknown, n (%)**	344 (0.01%)	217 (0.01%)	127 (0.02%)
